# Improving access to school health services as perceived by school professionals

**DOI:** 10.1186/s12913-017-2711-4

**Published:** 2017-11-17

**Authors:** Janine Bezem, Debbie Heinen, Ria Reis, Simone E. Buitendijk, Mattijs E. Numans, Paul L. Kocken

**Affiliations:** 1 Department of Preventive Youth Health Care, Municipal Health Service Gelderland-Midden, P.O. Box 5364, 6802 EJ Arnhem, The Netherlands; 2 Department of Child Health, TNO, P.O. Box 3005, 2301 DA Leiden, The Netherlands; 3 Department of Public Health and Primary Care, Leiden University Medical Centre (LUMC), P.O. Box 9600, 2300 RC Leiden, The Netherlands; 4 Education Office, Imperial College London, London SW7 2AZ, London, United Kingdom

**Keywords:** School health services, Accessibility, Task-shifting, Health assessments, Preventive child health care, Specific needs

## Abstract

**Background:**

The organisation of health assessments by preventive health services focusing on children’s health and educational performance needs to be improved due to evolving health priorities such as mental health problems, reduced budgets and shortages of physicians and nurses. We studied the impact on the school professionals’ perception of access to school health services (SHS) when a triage approach was used for population-based health assessments in primary schools. The triage approach involves pre-assessments by SHS assistants, with only those children in need of follow-up being assessed by a physician or nurse. The triage approach was compared with the usual approach in which all children are assessed by physicians and nurses.

**Methods:**

We conducted a cross-sectional study, comparing school professionals’ perceptions of the triage and the usual approach to SHS. The randomly selected school professionals completed digital questionnaires about contact frequency, the approachability of SHS and the appropriateness of support from SHS. School care coordinators and teachers were invited to participate in the study, resulting in a response of 444 (35.7%) professionals from schools working with the triage approach and 320 (44.6%) professionals working with the usual approach.

**Results:**

Respondents from schools using the triage approach had more contacts with SHS and were more satisfied with the appropriateness of support from SHS than respondents in the approach-as-usual group. No significant differences were found between the two groups in terms of the perceived approachability of SHS.

**Conclusions:**

School professionals were more positive about access to SHS when a triage approach to routine assessments was in place than when the usual approach was used. Countries with similar population-based SHS systems could benefit from a triage approach which gives physicians and nurses more opportunities to attend schools for consultations and assessments of children on demand.

**Electronic supplementary material:**

The online version of this article (10.1186/s12913-017-2711-4) contains supplementary material, which is available to authorized users.

## Background

Cognitive performance and educational achievements in children benefit from good health and health-related behaviours [1, 2]. The integration of preventive health services in the education system helps to detect health problems in school children and furthers early interventions intended to improve health and, therefore, cognitive outcomes [3–7]. Health services available in schools (School Health Services, SHS) include prevention, early detection and intervention in the area of school children’s physical, social and - increasingly - mental health [3, 4].

Research shows that equal access to SHS and SHS quality need to be improved for all groups of children. Furthermore, SHS should be tailored to health priorities such as overweight and mental health [4, 8, 9]. Health system issues relating to staff shortages in SHS, high workloads and inadequate demarcation of the position and responsibilities of SHS in educational institutions need to be addressed [4, 10].

These health system issues have challenged SHS to find innovative models which allow an efficient delivery of health services to school age children. In the Netherlands, community-based SHS professionals usually visit schools a few times a year to carry out routine assessments based on a pre-defined age schedule in which children between the ages of four and eighteen receive four routine health assessments from SHS physicians and nurses, sometimes supported by SHS assistants.

Physicians conduct the assessment in the youngest age group. Nurses conduct the assessments for older age groups, an approach that is also common in many other countries. The SHS delivers services free of charge. Interdisciplinary collaboration between professionals in the health-care and educational systems is organised in both approaches in multi-disciplinary school-based networks based on shared competencies, roles and responsibilities.

A novel approach was developed to conduct the routine health assessments based on triage and task-shifting among SHS professionals [11]. In this two-step triage procedure, pre-assessments were delegated to SHS assistants who had received specific training. Only children in need of follow-up were assessed by a SHS physician or nurse, which led to less involvement of physicians and nurses in the routine assessments. This created time for physicians and nurses to visit schools regularly and provide additional consultations that were tailored to children’s specific needs in response to requests from school professionals, parents and children themselves.

Triage and the shifting of tasks between health-care professionals have been used primarily in primary health care and emergency health-care services worldwide. Research shows that triage and task-shifting have several benefits: the optimal use of the skills and expertise of health-care professionals, reduced workloads for physicians and nurses, improved access to health care and greater patient satisfaction [12–15]. A pilot study examining the triage approach in SHS showed equal attendance levels of about 90% for health assessments in a comparison of the triage and usual approaches. Fewer children were referred for extra assessment by SHS or for treatment by external health services in the triage approach [11]. Another study showed that routine health assessments in a triage approach seemed to detect health problems as effectively as the usual approach [16].

The aim of this study was to explore how school professionals in primary schools experience access to population-based SHS systems when a triage approach is used for routine health assessments. We compared these perceptions with those of school professionals working with the usual SHS approach. In this study, school professionals are primary school teachers and care coordinators. The latter are teachers who also support children with specific needs.

We studied the views of school professionals about access to SHS because we know accessibility affects health service utilisation, consumer satisfaction and the quality of care [17–20]. Accessibility factors relating to health services include approachability, acceptability, availability, affordability and appropriateness of care [19]. The triage system specifically targeted the improvement of two aspects of access to preventive health services: approachability and the appropriateness of care. ‘Approachability’ refers to consumers’ ability to gain access to the service and to identify the existence of some form of service, and the terms also refers to the fact that a service can be reached, and the fact that it has an impact on health. ‘Appropriateness’ of care relates to the adequacy of the health services provided and this is linked to the willingness to use the services [19]. Acceptability, availability and affordability are less relevant for preventive services like SHS, which should be offered to whole populations of children proactively. This manuscript addresses the following research question: what is the impact on the school professionals’ perception of the approachability and appropriateness of SHS support for primary-school children when the triage approach is used rather than the usual approach?

## Methods

We conducted a cross-sectional survey of access to SHS as perceived by school professionals (in other words, as stated above, primary school teachers and care coordinators). We compared school professionals who had worked with the triage approach in SHS and those who had worked with the usual approach.

**Fig. 1:**
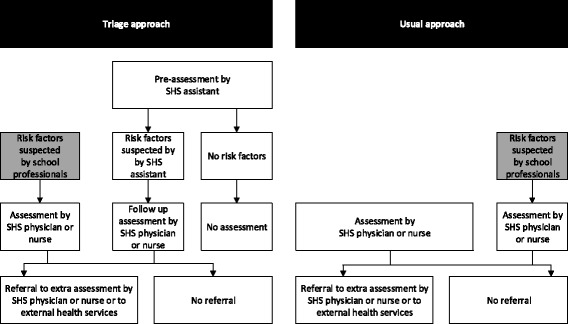
Process of routine health assessments by school health services (SHS); triage and usual approach

In the triage approach, SHS assistants follow a strict pre-assessment protocol to preselect children. The pre-assessment of the children is carried out based on: SHS records, questionnaires completed by school teachers and parents, and face-to-face screening. The assistants prioritise referral to SHS for children with suspected health-care needs. The next step is a follow-up assessment by a physician or nurse. Pre-assessment and follow-up assessments are part of the triage assessment procedure (see Fig. 1). In the usual approach to routine assessments, a physician or nurse assesses all children. In the triage and usual approaches, school professionals can refer children with suspected risk factors for an assessment by a SHS physician or nurse.

### Participants

Four distinct urban and non-urban areas in the Netherlands participated in the study. An urban and a non-urban area were selected for the triage and the usual approaches. One triage SHS had recently introduced the triage approach and the other SHS had done so five years before the study began. The two SHS in the approach-as-usual group had been working with this approach for a long time.

A two-step procedure was completed to select the study population. In the first step, 600 primary schools from the four geographical SHS regions were selected at random. In the second step, the school care coordinators and teachers in four school years (Kindergarten and school years 1, 4 and 6 (US system): children aged 5, 6, 9 and 12 years respectively) were selected from every school for inclusion in the study (1249 employees for the triage approach and 729 for the usual approach). Schools specifically for children with special needs, school professionals who had worked for less than six months at the school, and professionals other than teachers or care coordinators were excluded from the study. To ensure adequate power in the data analysis, we adopted a predefined significance level of 5% and statistical power of 80%. With a total population of about 1400 schools (in other words, all schools in the four SHS regions that were eligible for participation in the study), the minimum sample size was 300 schools. Assuming a school response rate of 50%, a sample of 600 schools was enough to ensure adequate power.

### Data collection

Data were obtained using a digital questionnaire sent by e-mail. Firstly, professionals at schools known to the SHS were approached to obtain the e-mail addresses of the respondents. In the triage group, e-mail addresses for most of the school care coordinators were available to the SHS. In the approach-as-usual group, we contacted school heads to obtain the e-mail addresses of the respondents. To maximise the response rate, a pre-notification letter was sent by e-mail to the school board and two reminders were sent to non-respondents [21]. The questionnaires were sent and returned in June and July 2012. Additional information about topics in terms of involvement in school networks or school size was collected through online searches.

The questionnaire items were based on the Consumer Quality Index [22, 23]. The concepts in this index were translated for use in this study. The questionnaire was developed with an expert group of SHS professionals. The questionnaire was pre-tested in a group of six school care coordinators. This resulted in only small changes in the wording of sentences and word selection.

This is a general accepted procedure when questionnaires tailored to the study group are not available.

Three scales were established to measure school professionals’ perceptions of access to SHS: two scales for approachability and one for the appropriateness of SHS support (see Table 1; for a full overview of the questions and the scales, see Additional file 1). The two scales for approachability were: a five-item scale ‘SHS approachability for contact and feedback’ and a two-item scale ‘SHS approachability for support for health issues’. In addition, a three-item scale ‘appropriateness of SHS support for children with specific needs’ was established. The answer categories used five-point Likert scales for the statements (ranging from ‘strongly disagree’ to ‘strongly agree’) or four-point Likert scales for the questions (ranging from ‘never’ to ‘always’). A question about the number of contacts between the school and SHS professionals was added to measure the contact frequency between the school and SHS in the previous six months. The answer categories were 0, 1–2, 3–4, 5–6 and >6 times.

**Table 1:** Questionnaire item/scales measuring how school professionals perceive access to school health services (SHS)

	Number of questions	Cronbach’s alpha (*α*) or Pearson correlation (*r*)	Example of questions, answer categories and score range
Item			
Contact frequency between school and SHS	1	–	How often did you have contact with SHS professionals in the last six months in addition to the regular assessments? 0 times (1) more than six times (5) (5 categories)
Scales			
SHS approachability for contact and feedback	5	*α* = 0.79	Can you reach SHS professionals when you need them? never (1) always (4) (5 categories including not applicable)
SHS approachability for support for health issues	2	*r* = 0.64	To what extent do you agree or disagree with the statement: I contact SHS when I have concerns about a pupil’s health: strongly disagree (1) strongly agree (5) (6 categories including no opinion)
Appropriateness of support provided by SHS for children with specific needs	3	*α* = 0.74	To what extent do you agree or disagree with the statement: SHS ensures children with specific needs are referred to proper care in time: strongly disagree (1) strongly agree (5) (6 categories including no opinion)

The demographic and descriptive data relating to schools and school professionals were collected using the questionnaire and online searches of the schools’ characteristics, including the involvement of SHS in school-based networks, school size, municipality size, position of the school professional (teacher or school care coordinator), and number of years working at the current school. The socio-economic status of the school population was determined on the basis of the postal codes of the schools and was based on education, income and employment status of the inhabitants of the school area.

### Data analysis

We used descriptive analyses and chi-square tests to assess differences between the background characteristics of schools and school professionals in either the triage group or the approach-as-usual group.

Scales were constructed using multiple steps. At first, we converted the answer categories of the ordinal variables into quantified (continuous numeric) variables for all subsequent analyses using categorical principal component analysis (for further details, see [24]). Secondly, the quantified variables were clustered into scales using a principal component analysis. The discriminant validity of the scales was tested using the eigenvalues of the factors and the associated scree plot. According to the Guttman-Kaiser criterion, factors with an eigenvalue greater than one were retained. Thirdly, drawing on research [19, 20] and the principal component analysis, we constructed three scales: ‘SHS approachability for contact and feedback’, ‘SHS approachability for support for health issues’ and ‘appropriateness of SHS support for children with specific needs’ (see Table 1). The reliability of the scales was analysed using Cronbach’s alpha coefficients or Pearson’s correlation coefficients.

Our next step was to analyse the differences between school professionals’ perceptions of access to SHS in the triage and approach-as-usual conditions using multilevel regression analysis with contact frequency, the approachability of SHS and the appropriateness of support as outcome variables and the approach (triage or usual approach) and differences between background characteristics as the independent variables. A multilevel regression analysis was required because of the lack of independence between school professionals in individual schools [25, 26]. The size of the differences between the triage approach and usual approach was given using standardised regression coefficients with 95% confidence intervals. SPSS Statistics was used to analyse the data (SPSS 21.0 for Windows, SPSS Inc., Chicago, IL).

## Results

**Fig. 2:**
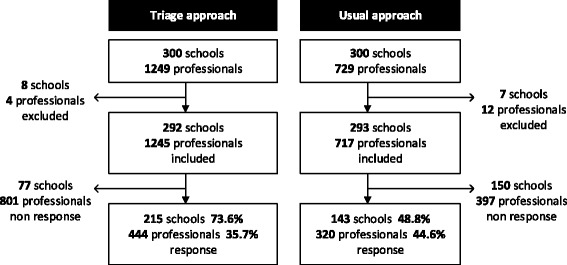
Response flow diagram; triage and usual approach

Figure 2 shows the questionnaire responses for the triage and usual approach. Data relating to four school professionals and eight schools in the triage group, and twelve school professionals and seven schools in the usual approach group, had to be excluded due to non-conformity with our inclusion criteria or because the professionals could not be reached due to an incorrect e-mail address.

The response rate was 73.3% for the schools in the triage group and 48.8% for the schools in the approach-as-usual group. The response from the professionals was 35.7% in the triage group and 48.6% in the approach-as-usual group (see Fig. 2). The most frequently stated reason for not participating in the study was a lack of time.

Our data showed a difference between the schools using the two approaches in terms of the municipality size (Table 2). Schools in the triage group were situated less often in municipalities with fewer than 40,000 residents than schools in the approach-as-usual group. No other differences were found between the triage and approach-as-usual group in terms of the background characteristics of schools such as the participation of SHS in school-based networks, the socio-economic status of the school population or school size.

**Table 2:** Characteristics of schools and respondents; triage and usual approach

	Triage approach	Usual approach	Total group
	*N* (%)	*N* (%)	*N* (%)
School characteristics	*N* = 215	*N* = 143	*N* = 358
Schools with SHS participating in interdisciplinary network			
No	28 (16.9)	20 (16.4)	48 (16.7)
Yes	138 (83.1)	102 (83.6)	240 (83.3)
Missing	49	21	70
Socio-economic status of the school population			
Low	77 (36.0)	49 (34.8)	126 (35.5)
Middle	90 (42.1)	60 (42.6)	150 (42.3)
High	47 (22.0)	32 (22.7)	79 (22.3)
Missing	1	2	3
School size			
≤ 200 pupils	104 (56.5)	73 (58.9)	177 (57.5)
> 200 pupils	80 (43.5)	51 (41.1)	131 (42.5)
Missing	31	19	50
Municipality size^*^			
≤ 40,000 residents	97 (45.3)	87 (61.7)	184 (51.8)
> 40,000 residents	117 (54.7)	54 (38.3)	171 (48.2)
Missing	1	2	3
Respondent characteristics	*N* = 444	*N* = 320	*N* = 764
Position school professional			
School care coordinator	134 (30.2)	81 (25.3)	215 (28.1)
Teacher	310 (69.8)	239 (74.7)	549 (71.9)
Number of years working at the current school			
½ - 1	23 (5.2)	16 (5.0)	39 (5.1)
1–5	105 (23.6)	72 (22.5)	177 (23.2)
5–10	91 (20.5)	48 (15.0)	139 (18.2)
≥ 10	225 (50.7)	184 (57.5)	409 (53.5)

^*^ *p* < 0.01

Our study showed a difference in contact frequency and school professionals’ perceptions of the appropriateness of SHS support between the two study groups (triage and usual approach) (Table 3). The school professionals in the triage group reported significantly more contact with SHS professionals than professionals in the approach-as-usual group. In addition, we found differences in perception with respect to the appropriateness of support provided by SHS professionals. More school professionals in the triage group than in the approach-as-usual group thought the support provided by SHS for children with specific needs was appropriate. The main difference between the schools relates to the scale item ‘SHS makes an important contribution to the detection of problems’. The values of the standardised betas in Table 3 reflect the strength of the measured relationship. The association between the SHS approach and the frequency of contact between the school and SHS is stronger than the association with the appropriateness of support provided for children with specific needs.

**Table 3:** School professionals’ perceptions about access to school health services (SHS); triage and usual approach

	Triage approach *N* = 444	Usual approach *N* = 320	
	Mean (SD)^a^	Mean (SD)^a^	β (95% CI)^b^
Item			
Contact frequency between school and SHS^c^	1.71 (0.99)	1.41 (0.74)	0.26 (0.13–0.39)*
Scales^d^			
SHS approachability for contact and feedback	−0.02 (0.73)	0.02 (0.74)	−0.02 (−0.14–0.10)
SHS approachability for support for health issues	0.00 (0.86)	0.00 (0.97)	0.00 (−0.14–0.14)
Appropriateness of provided SHS support of children with specific needs	0.05 (0.81)	−0.07 (0.80)	0.13 (0.01–0.20)**

*SD* Standard Deviation; β = standardized regression coefficient; CI = Confidence Interval

*p < 0.001; **p < 0.05

^a^Crude mean values

^b^Multilevel regression analyses with the scales and contact frequency as outcome variables and the approach (triage and usual) and municipality size as independent variables. Standardized regression coefficient (β) and confidence interval (CI)

^c^The contact frequency was measured on a five-point scale with answer categories 0, 1–2, 3–4, 5–6 and >6 times (coded 1 to 5)

^d^All categorical variables of the scales were converted to quantified (i.e. continuous numeric) variables with a mean of 0 (using categorical principal component analysis). A higher value means more satisfaction for the scale described

No impact was found on the perceived approachability of SHS evidenced by the response from school professionals on the scales ‘SHS approachability for contact and feedback’ and ‘SHS approachability for support for health issues’ in the comparison of the triage group with the approach-as-usual group.

## Discussion

The aim of this study was to explore how school professionals in primary schools experience working with a triage approach to the routine assessments conducted by school health services (SHS) and to make a comparison with school professionals who were offered the usual SHS approach. An difference was found between the two groups in the perceived appropriateness of support from SHS and the contact frequency between school and SHS professionals. These differences may be linked to the differences between the two approaches. In both approaches, SHS professionals visit schools both using a predefined schedule and when necessary. A triage assessment procedure creates time for physicians and nurses to visit schools regularly to conduct additional assessments of children with specific needs when asked to do so by school professionals, parents and children themselves. The procedure also creates more possibilities to cooperate in school-based networks. The triage approach contributes to the sharing of information between school and health professionals about children with specific needs and the early detection of health problems, and this may explain the positive evaluation of the appropriateness of SHS support. On the other hand, it is possible that children are missed in the assessments by assistants in the triage approach, and parents are less involved in the first step of the triage procedure. However, school professionals are in contact with almost every child daily, making it possible to identify children with health-related problems. Other studies show that the efficiency and responsiveness of the health care system are known to be linked to the approachability and expertise of health-care professionals [12, 27, 28]. The perceived approachability of SHS will not have changed because dedicated professionals are active in the Dutch SHS system in both approaches.

### Strengths and limitations

A strength of our study is that we sent questionnaires to a random sample of schools. Respondents completed the digital questionnaires anonymously and this may have improved the reliability of the results. The background characteristics of the study groups were similar, except for the municipality sizes, which we corrected for in the analyses.

A methodological limitation is the low response rates, although this is not uncommon in surveys of both schools and professionals in those schools. The results may have been positively affected by the higher response rates from schools and school professionals who were positive about access to SHS. We expect schools and school professionals who are satisfied with either approach or who have had more contact with SHS professionals to be more willing to participate in the study. This would imply an overestimation of the findings for our outcome measures, appropriateness of care and contact frequency.

A difference in the response rates was found between the schools. We found that more schools located in relatively larger municipalities in the triage group responded than schools in those municipalities in the approach-as-usual group. Although we corrected for this in the analyses, the higher scores for appropriateness and contact frequency may suffer from bias due to the more frequent and severe health problems in children living in a more urban area, leading to more SHS activities. On the other hand, there were no differences between the schools in terms of socio-economic status. Because this is an important factor for the health status of children and the correlation with urbanisation, we expect that differences in levels of urbanisation to have a minor effect in schools using the triage and usual approaches.

We were not able to analyse differences between the characteristics of schools or professionals in the response and non-response groups because the background characteristics of the non-response group were not available.

Another possible cause of bias is that the outcomes of the triage approach may have been affected by the fact that the triage approach had not been in place for as long as the usual approach. The professionals using the triage approach have less experience with this novel method, and this could lead to contact frequency and the appropriateness of care being underestimated. Triage can reasonably be expected to have a stronger impact on appropriateness of care and possibly on approachability when it has been in place for a longer period of time.

Finally, we used a self-report questionnaire based on an existing instrument to measure school professionals’ perceptions of approachability and the appropriateness of support from SHS. Further questionnaire development and research into the validity of the questionnaire are recommended.

### Implications for school health services

SHS systems are available in many countries, and they are often delivered by nurses. The efficiency and quality of these SHS systems need to be optimised to improve children’s health and development and to tailor SHS to school systems [4, 10]. A change in the organisational model of SHS is needed for the efficient use of resources available in the system and to solve the problem of a shortage of SHS physicians and nurses. Most countries with a high level of staffing have introduced reforms in the last five years, including triage and task-shifting [4]. The benefits of task-shifting are already widely known in health care. We studied the impact of introducing triage and task-shifting to SHS because there has not yet been enough research in this field of health care. Our study showed that the use of a triage approach by SHS could be advisable in countries with similar population-based SHS systems involving routine assessments conducted by physicians and nurses. The involvement of assistants in the routine assessments could improve the efficiency of SHS. A triage approach used by population-based SHS systems could create opportunities for nurses and physicians to increase the contact frequency with schools to deliver care on demand and to enhance the collaboration and relationship between school and health professionals. This improved collaboration between schools and health professionals is expected to contribute to the early detection of health-related school problems and to benefit children with specific needs.

### Implications for research

An examination of more objective outcome measures such as lower school absenteeism for the support provided by schools and SHS professionals for children with specific needs is advised to enhance our understanding of the benefits of investment in collaboration between the two systems. Further research is also recommended into the position and responsibilities of SHS and school professionals with a view to improving collaboration between the two systems to improve children’s health and well-being. Parents’ experiences with the triage approach represent another area requiring study.

## Conclusions

School professionals had more contacts with SHS professionals and were more positive about the appropriateness of support from SHS when a triage approach to routine assessments was in place than when the usual approach was used. Countries with similar population-based SHS systems could benefit from a triage approach which gives physicians and nurses more opportunities to attend schools for consultations and assessments of children on demand.

## Additional file

Additional file 1: Questions and scales in the online questionnaire. Description of questions, answer categories and score range in the online questionnaire. (DOCX 12 kb)

## Abbreviation

SHS: School Health Services
