# Supplementary material for: Improving access to school health services as perceived by school professionals
Source: BMC Health Serv Res. 2017 Nov 17;17:743. doi: 10.1186/s12913-017-2711-4 (PMC5693589; doi:10.1186/s12913-017-2711-4)
Supplement: Additional file 1: — Questions and scales in the online questionnaire. Description of questions, answer categories and score range in the online questionnaire. (DOCX 12 kb) [file 12913_2017_2711_MOESM1_ESM.docx]

|  | Questions, answer categories and score range |
| --- | --- |
| **Item** | |
| Contact frequency between school and SHS | How often did you have contact with SHS professionals in the last six months in addition to the regular assessments? 0 times (1) more than six times (5) (5 categories) |
| **Scales** | |
| SHS approachability for contact and feedback | Can you reach SHS professionals when you need them? never (1) always (4) (5 categories including not applicable) |
|  | Do you receive a response in time from SHS to your questions about children with specific needs? never (1) always (4) (5 categories including not applicable) |
|  | Are you satisfied with the number of contacts with SHS professionals? no (1) yes (2) (2 categories) |
|  | Do you receive useful feedback from SHS on your questions about children with specific needs? never (1) always (4) (5 categories including not applicable) |
|  | Do you receive sufficient feedback from SHS after the health assessments? never (1) always (4) (5 categories including no opinion) |
| SHS approachability for support for health issues | To what extent do you agree or disagree with the statement: I contact SHS when I have concerns about a pupils’ health: strongly disagree (1) strongly agree (5) (6 categories including no opinion) |
|  | To what extent do you agree or disagree with the statement: I contact SHS when I have concerns about a pupils’ psychosocial development: strongly disagree (1) strongly agree (5) (6 categories including no opinion) |
| Appropriateness of provided SHS support for children with special needs | To what extent do you agree or disagree with the statement: SHS provides an important contribution in detecting problems: strongly disagree (1) strongly agree (5) (6 categories including no opinion) |
|  | To what extent do you agree or disagree with the statement: SHS ensures children with specific needs are referred to proper care in time: strongly disagree (1) strongly agree (5) (6 categories including no opinion) |
|  | To what extent do you agree or disagree with the statement: SHS gives sufficient attention to children with specific needs: strongly disagree (1) strongly agree (5) (6 categories including no opinion) |
